# Non-surgical treatment of peri-implantitis with submarginal erythritol air-polishing in severe periodontitis patients: a randomized controlled trial

**DOI:** 10.1186/s12903-026-07721-z

**Published:** 2026-01-19

**Authors:** Shiai Dai, Xianghui Feng, Xuyang Gao, Dong Shi

**Affiliations:** 1 Department of Periodontology, Peking University School and Hospital of Stomatology, Beijing, China; 2 Peking University School of Stomatology, No.22, Zhongguancun South Avenue, Haidian District, Beijing, China

**Keywords:** Dental implant, Peri-implantitis, Erythritol air-polishing, Non-surgical treatment, Periodontitis, Randomized controlled trial

## Abstract

**Aim:**

To evaluate the clinical and radiographic outcomes of non-surgical treatment by either submarginal erythritol air-polishing or combined instrumentation for peri-implantitis in severe periodontitis patients.

**Materials and methods:**

This randomized clinical trial enrolled patients diagnosed with stage III/IV periodontitis and peri-implantitis affecting at least one dental implant. The test group received erythritol air-polishing while the control group received ultrasonic combined with manual instrumentation for peri-implant submarginal debridement. The primary outcome was the mean peri-implant probing depth (PPD) at 3 months. Clinical parameters were assessed at baseline (T0), T1 (6 weeks), and T2 (3 months); radiographic parameters were assessed at T0 and T2. Intergroup differences were analyzed using the Mann-Whitney U or t-test at each timepoint and verified by mixed-effects models. Factors potentially influencing PPD prognosis were assessed using Generalized Estimating Equations (GEE).

**Results:**

Initially, 40 patients (20 per group) with peri-implantitis were randomly assigned to the trial; 38 patients completed the study. At T2, mean PPD reduced from 6.0 ± 1.8 mm to 4.2 ± 1.6 mm (test group) and 4.7 ± 1.5 mm (control group) with no significant differences between groups. Mean modified sulcus bleeding index(mSBI) decreased in both groups, with a significantly greater reduction in the test group. The treatment efficacy of PPD can be influenced by keratinized mucosa width (KMW), PPD-T0, mSBI-T2, and probing depth reduction of adjacent teeth.

**Conclusions:**

In patients with severe periodontitis, both non-surgical therapies were effective in improving clinical parameters of peri-implantitis. Erythritol air-polishing exhibited greater improvement in soft tissue inflammation. Adequate peri-implant keratinized mucosa and greater reduction in probing depth of adjacent teeth were identified as favorable prognostic factors for PPD.

**Clinical trial registration:**

This study had retrospectively registered at Chinese Clinical Trial Registry (ChiCTR2100048446) on July 7, 2021 (https://www.chictr.org.cn/showproj.html? proj=129220).

## Introduction

 Peri-implantitis is defined as a pathological condition occurring in tissues around dental implants, characterized by inflammation in the peri-implant connective tissue and progressive loss of supporting bone [1], with the patient level prevalence of 25% (21.1–29.3%) and an implant level prevalence of 18% (15.8–20.5%) [2]. Etiologically, peri‑implantitis is initiated by colonization of microbial biofilms on implant surfaces. In addition, multiple risk factors are associated with its development. Among these, a history of severe periodontitis is strongly supported by current evidence as a major risk factor [1]. Compared to no/mild periodontitis individuals, patients with severe periodontitis demonstrate substantially elevated peri-implant disease susceptibility [3–6]. Furthermore, the stage of periodontitis directly correlates with peri-implant attachment loss and bone resorption [7–9].

For the management of peri-implantitis, a step-by-step approach is recommended. Non-surgical therapy should be the initial intervention, which primarily involves the use of supramarginal and submarginal instruments to remove local contributing factors (e.g., biofilm, calculus, and residual cement). Due to the factors such as implant thread morphology, complex peri-implant infrabony defects and altered surface roughness induced by conventional instrumentation, debridement around implants is particularly difficult and the therapeutic effect is unpredictable [10]. Non-surgical debridement relies on mechanical/physical modalities including sonic/ultrasonic devices, manual instruments (scalers/curettes), air-polishing, and light-mediated therapies (lasers/photodynamic therapy) [11–14]. To date, effective control of peri-implantitis remains suboptimal, particularly in advanced cases [14]. Therefore, air-polishing emerges as a viable alternative, utilizing a high-velocity stream of a water and abrasive powder mixture to effectively remove dental plaque from implant surfaces [15]. Erythritol air-polishing powder debuted in 2013, which is smaller (diameter of 14 μm) and harder than glycine powder. In addition, it contains 0.3% chlorhexidine. The clinical evidence of the effectiveness of erythritol air‑polishing in the non-surgical treatment of peri-implantitis is limited. Some studies have used it as an adjunct [16, 17], and standalone use is rarely reported [18, 19], especially in patients with severe periodontitis. Despite potential benefits, air‑polishing may also carry certain risks, including subcutaneous emphysema [20–22]. Therefore, the efficacy and safety of erythritol air‑polishing for non-surgical treatment of peri-implantitis require further evaluation through clinical studies.

This randomized controlled clinical trial aims to test the hypothesis that erythritol air-polishing achieves equivalent clinical and radiographic outcomes to ultrasonic combined with manual instrumentation for non-surgical management of peri-implantitis in patients with stage III/IV periodontitis.​​.

## Materials and methods

### Study design and ethical statements

This trial was designed as a randomized controlled clinical trial with two parallel groups and a follow-up of 3 months, which was conducted at the Department of Periodontology of Peking University School of Stomatology from 2022 to 2024 in accordance with the Declaration of Helsinki​ (1975 version, revised 2013). Ethical approval was obtained from the Ethics Committee of Peking University School of Stomatology (PKUSSIRB-202056099). Reporting followed the Consolidated Standards of Reporting Trials (CONSORT) 2010 guidelines [23]. All patients signed an informed consent before enrollment.

### Study population

​ Patients diagnosed with stage III/IV periodontitis and peri-implantitis affecting at least one dental implant were enrolled.​​ Periodontitis and peri-implantitis was diagnosed following the case definition of the 2017 World Workshop on the Classification of Periodontal and Peri-Implant Diseases and Conditions, with corresponding criteria applied to implants (with or without initial examination) based on the most recent follow-up records [1, 24]. The other following inclusion criteria were applied: (1) Age 18 to 75 years; (2) At least one dental implant diagnosed with peri-implantitis and at least one site presenting probing depth ≥ 6 mm​; (3) Implant‑supported fixed prosthesis functionally loaded for > 1 year; (4) If multiple eligible implants existed, only the implant with the deepest probing depth was enrolled​​. Furthermore, the following exclusion criteria were applied: (1) Systemic diseases affecting treatment efficacy (e.g., diabetes, osteoporosis, use of bisphosphonates or anticoagulants); (2) Respiratory diseases (e.g., chronic bronchitis, asthma); (3) Current smokers or those who quit < 5 years ago; (4) Antibiotic use within 3 months or periodontal/peri-implant maintenance therapy within 6 months; (5) Obvious submarginal calculus or residual adhesives detectable on implant surfaces via probing or periapical radiographs.

### Interventions

#### Pre-randomization interventions

The enrolled patients received a comprehensive periodontal examination of both natural teeth and implants. Before the intervention, all patients received oral hygiene instruction and initial periodontal therapy of all natural teeth. Supramarginal cleaning of dental implants was performed with PEEK tips and rubber cups. Before peri-implantitis therapy was initiated, the full-mouth plaque score (FMPS) in all patients were controlled below 25% [25].

#### Post-randomization interventions

After ​randomization of all enrolled patients, implants diagnosed with peri-implantitis received different therapies according to the appropriate grouping. All treatment was performed by the same experienced periodontist (X.H.F.). Anesthesia was administered as needed. During treatment, all implant prostheses were not removed (two implant prostheses removed prior to the visit in each group). Test group received submarginal air-polishing of the implant using erythritol powder (PLUS^®^ powder, Electro Medical Systems (EMS), Switzerland) with a nozzle inserted on six sites of the implant, parallel to the implant surfaces, with 5 s of treatment per site (mesio-buccal, mid-buccal, disto-buccal, mesio-lingual, mid-lingual, and disto-lingual). Control group received submarginal scaling with the piezoelectric ultrasonic scaler with a PEEK-coated plastic tip (PI instrument, EMS, Switzerland) and manual titanium curettes (LM^®^, Finland) to achieve implant debridement for at least 30 s. All implant sites received submarginal irrigation with 0.12% chlorhexidine acetate solution immediately after treatment completion in both groups. All patients did not be permitted to take antibiotics during the study. At each follow-up visit, the periodontist reinforced oral hygiene instruction to patients.​.

### Clinical examination

Clinical assessments were performed at baseline (T0, 1 week after supramarginal cleaning of the implant), 6 weeks (T1) and 3 months (T2) after submarginal debridement of peri-implantitis. All clinical examinations were conducted by a professional examiner (D.S.), who received standardized training on the study protocol and measurement techniques. Prior to the data collection, the examiner performed repeated measurements of probing examination on 9 implants and reliability was confirmed. Good intra‑examiner reliability was demonstrated, with an intraclass correlation coefficient (ICC) of 0.93.

Probing examination was performed on each implant using a stainless steel Williams probe (Shanghai Kangqiao Dental Instrument Factory, China) with a light force. Clinical examination of implants included: (1) modified plaque index (mPlI) [26]; (2) peri-implant probing depth (PPD), the distance (mm) from the mucosal margin to the bottom of the periodontal pocket; (3) modified sulcus bleeding index (mSBI) [26]; (4) presence or absence of suppuration [27]; (5) peri-implant clinical crown length (PCCL), the distance (mm) from crown top edge to mucosal margin; (6) keratinized mucosa width (KMW) at the mid-buccal site, the distance (mm) from the mucosal margin to the mucogingival junction; (7) peri-implant mucosal thickness (MT), classified into thick/thin using ​transgingival probe visibility method [28]. Except for the KMW and MT, other clinical parameters were assessed at six sites of each implant. Clinical examination of natural teeth included: (1) probing depth (PD); (2) bleeding index (BI) [29], with BI scores ≥ 2 indicating positive bleeding on probing (BoP). Additionally, patient-related data (age, sex, periodontitis stage, compliance with periodontal maintenance, oral hygiene practices, bruxism), implant-related parameters (post-implant time, location, implant system), and prosthesis-related characteristics (retention connection, restoration cleanability) were documented at baseline.​​.

### Radiographic examination

Periapical radiographs were taken at T0 and T2, which were then independently measured by two examiners (S.A.D. and X.Y.G.) using the Geometer’s Sketchpad V5.01, and the mean value was taken as the final result. Before the study, the examiners underwent calibration training and performed duplicate measurements on 20 sample radiographs. Inter‑examiner agreement was assessed, with a final ICC = 0.91. All radiographs were calibrated using the known length of the implant, with measurements accurate to 0.1 mm. The marginal bone level (MBL) was defined as the distance from a reference point on the implant (the junction of the implant neck’s smooth incline and the microthread) to the most coronal point of contact at the implant-bone interface.

### Outcomes

Treatment success at T2 was defined according to the 2023 EFP Clinical Practice Guideline and included the following criteria [30]: (1) PPD ≤ 5 mm; (2) no suppuration; (3) no BOP (mSBI ≥ 1) at more than one site.

The primary outcome was the mean value of PPD at T2. Secondary outcomes included the assessment of (1) success rate; (2) deepest PPD (PPDd, deepest pocket recorded in six sites for each implant); (3) mSBI (mean of six sites); (4) mPlI; (5) suppuration; (6) mucosal recession (REC, calculated as the mean difference in PCCL); (7) MBL.

### Sample size

With PPD as the primary endpoint, a 1 mm group difference was considered clinically relevant based on the minimal clinically important difference (MCID), with an assumed standard deviation of 1 mm. A two-sided significance level (α) of 0.05 and 85% statistical power (1-β) were set. Sample size estimation using the t-test function in PASS V21.0.4 indicated a minimum requirement of 18 participants per group. To account for a potential 10% attrition rate, the sample size was adjusted to 20 per group, resulting in a total of 40 participants.

### Groups, randomization and blinding

Patient randomization was computer-generated using the random number table by a blinded independent researcher (X.Y.G.). Blinding was maintained for the operator, examiner, and statistician throughout the study.​​.

### Data collection and statistical analysis

Statistical analyses were conducted in SPSS V.27.0 and R 4.3. Categorical variables were analyzed by chi-square or Fisher’s exact tests. Normality of quantitative variables was assessed via the Shapiro-Wilk test and histogram inspection. Depending on distribution, intergroup differences were evaluated using independent t-tests (normal) or Wilcoxon rank-sum tests (non-normal). Intragroup temporal changes were assessed via paired t-tests (normal) or Wilcoxon signed-rank tests (non-normal). For repeated-measures data (e.g., PPD, mSBI, mPlI measured at six sites per implant at T0/T1/T2), a three-level hierarchical structure was specified: measurements (level 1) nested within sites (level 2) nested within patients (level 3). Mixed-effects models accounted for intra-cluster correlations to obtain accurate estimates. The binary outcome variable “PPD-T2 ≤ 5 mm” was modeled using a two-level hierarchical structure via Generalized Estimating Equations (GEE) with logit link function to estimate covariate influences.​​ Statistical significance was set at a two-tailed *P* < 0.05.​​.

### Withdrawal

During therapeutic intervention, potential complications (e.g., subcutaneous emphysema in soft tissues) require immediate cessation of treatment and proper management. Patients retained the unconditional right to withdraw from this trial at any time.​​.

## Results

**Fig. 1:**
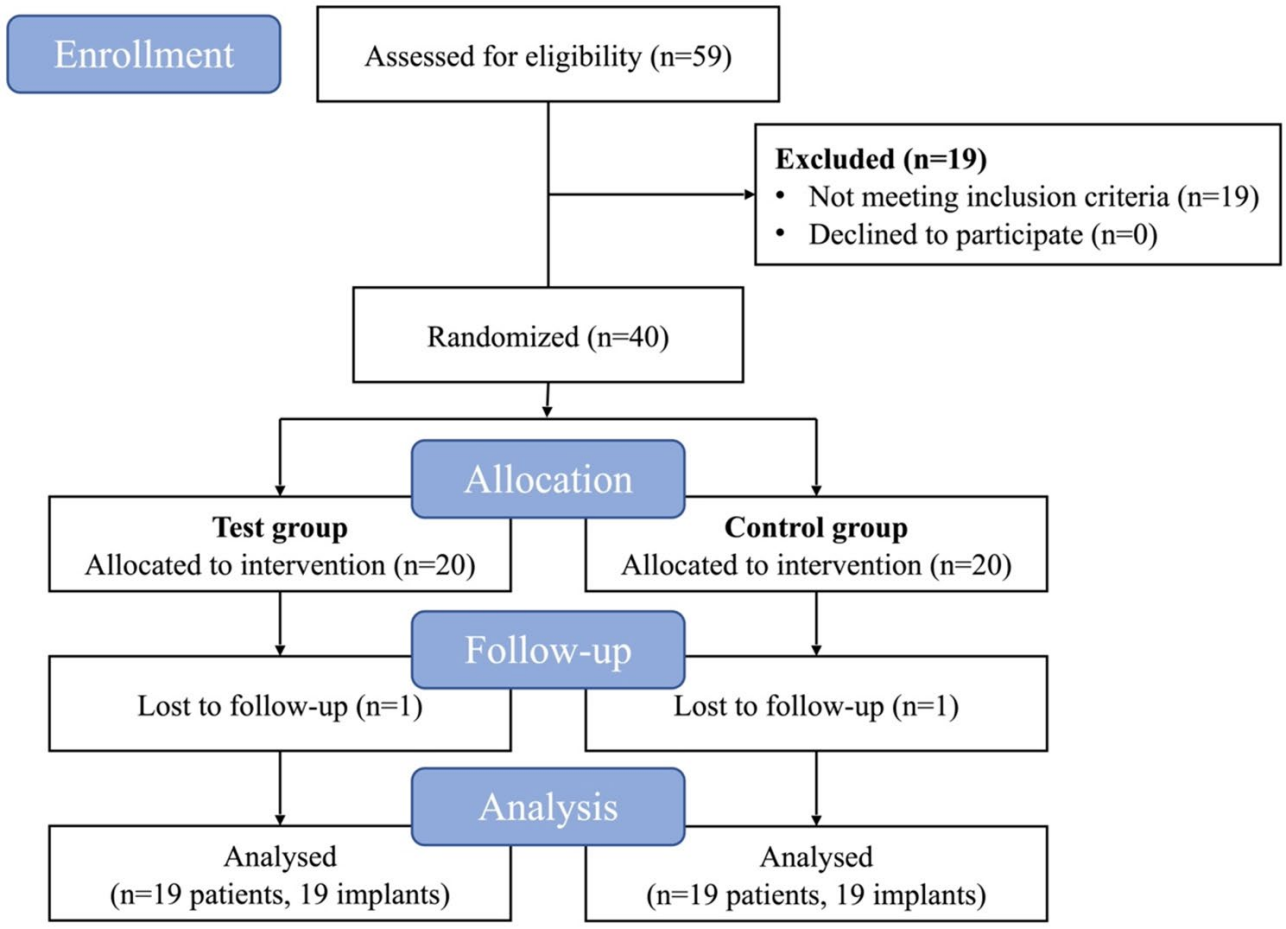
CONSORT flow diagram of the study

The study flowchart is presented in Fig. 1. Initially, 40 patients were enrolled. Two patients (one patient in each group) dropped out after debridement due to relocation and mobility constraints, resulting in 38 patients for final analysis. The characteristics of the patients and implants are detailed in Tables 1 and 2. At T0, no apparent imbalances existed between groups. No cases of emphysema or severe pain complications were reported during the treatment or follow-up.

**Table 1:** Patient characteristics

Variables			Groups		*P*
	Total (*n* = 38)		Test group (*n* = 19)	Control group (*n* = 19)	
Mean age,years	50.1 ± 10.0		47.4 ± 8.9	52.8 ± 10.5	0.095
Sex,*n*(%)					
Male	18(47.37)		8(42.11)	10(52.63)	0.746
Female	20(52.63)		11(57.89)	9(47.37)	
Stage of periodontitis,*n* (%)					
Stage III	20(52.63)		13(68.42)	7(36.84)	0.103
Stage IV	18(47.37)		6(31.58)	12(63.16)	
Discomfort symptoms,*n* (%)					
With	14(36.84)		6(31.58)	8(42.11)	0.737
Without	24(63.16)		13(68.42)	11(57.89)	
Periodontal treatment before implant surgery,*n* (%)					
Scaling and root planing	28(73.68)		13(68.42)	15(78.95)	0.546
Only supragingival scaling	7(18.42)		5(26.32)	2(10.53)	
None	3(7.89)		1(5.26)	2(10.53)	
Frequency of periodontal treatment after implant surgery,*n* (%)					
Every year	13(34.21)		7(36.84)	6(31.58)	0.696
1–2 years	10(26.32)		6(31.58)	4(21.05)	
≥ 3 years	6(15.79)		3(15.79)	3(15.79)	
None	9(23.68)		3(15.79)	6(31.58)	
Bruxism,*n* (%)					
Without	33(86.84)		16(84.21)	17(89.47)	1.000
With	5(13.16)		3(15.79)	2(10.53)	
Tooth brushing habit,*n* (%)					
≥ twice/day	37(97.37)		19(100.00)	18(94.74)	1.000
once/day	1(2.63)		0(0.00)	1(5.26)	
Interdental cleaning habit,*n* (%)					
≥ once/2 day	28(73.68)		15(78.95)	13(68.42)	0.246
Occasionally	6(15.79)		3(15.79)	3(15.79)	
Never	4(10.53)	1(5.26)		3(15.79)	

**Table 2:** Implant characteristics

Variables		Groups		*P*
	Total (*n* = 38)	Test group (*n* = 19)	Control group (*n* = 19)	
Post-implant time​				
Mean, years	7.59 ± 3.75	7.92 ± 4.12	7.26 ± 3.41	0.595
< 5 years, *n* (%)	7(18.42)	3(15.79)	4(21.05)	1.000
5–10 years, *n* (%)	31(81.58)	16(84.21)	15(78.95)	
Location,*n* (%)				
Maxilla	22(57.89)	12(63.16)	10(52.63)	0.743
Mandibula	16(42.11)	7(36.84)	9(47.37)	
Anterior tooth	12(31.58)	8(42.11)	4(21.05)	0.419
Premolar	6(15.79)	2(10.53)	4(21.05)	
Molar	20(52.63)	9(47.37)	11(57.89)	
Implant system,*n* (%)				
Straumann	17(44.74)	6(31.58)	11(57.89)	0.274
Bicon	7(18.42)	4(21.05)	3(15.79)	
Others	14(36.84)	9(47.37)	5(26.32)	
Crown/bridge connection,*n* (%)				
Screw-retained	16(42.11)	8(42.11)	8(42.11)	1.000
Cemented	22(57.89)	11(57.89)	11(57.89)	
Restoration cleanability,*n* (%)				
Easily	33(86.84)	16(84.21)	17(89.47)	1.000
Hard	5(13.16)	3(15.79)	2(10.53)	
MT,*n* (%)				
Thin	17(44.74)	6(31.58)	11(57.89)	0.191
Thick	21(55.26)	13(68.42)	8(42.11)	
PIKM,*n* (%)				
<2 mm	6(15.79)	2(10.53)	4(21.05)	0.660
≥ 2 mm	32(84.21)	17(89.47)	15(78.95)	

Abbreviations: *MT* mucosal thickness, *PIKM* peri-implant keratinized mucosa

### Primary outcome

Peri-implant clinical outcomes are presented in Table 3.​​ At T2, PPD decreased significantly from baseline (6.0 ± 1.8 mm) in both groups, but no statistically significant intergroup difference was observed (Table 4). Regarding change values, the test group showed greater improvement (1.8 ± 1.6 mm) than the control group (1.3 ± 1.6 mm).

**Table 3:** Peri-implant clinical parameters at baseline(T0), 6 weeks(T1) and 3 months(T2) results

	Groups	T0	T1	ΔT0-T1	T2	ΔT0-T2
Peri-implant clinical parameters						
Mean PPD, mm	Test Control Total	6.0 ± 1.9 6.0 ± 1.7 6.0 ± 1.8	4.4 ± 1.8 4.7 ± 1.6 4.5 ± 1.7	1.6 ± 1.4 1.3 ± 1.5 1.5 ± 1.5	4.2 ± 1.6 4.7 ± 1.5 4.4 ± 1.6	1.8 ± 1.6 1.3 ± 1.6 1.6 ± 1.6
Mean PPDd, mm	Test Control Total	7.5 ± 1.2 7.4 ± 1.0 7.5 ± 1.1	5.8 ± 1.5 5.8 ± 1.8 5.8 ± 1.7	1.7 ± 0.8 1.6 ± 1.5 1.6 ± 1.2	5.5 ± 1.4 5.8 ± 1.2 5.6 ± 1.3	2.1 ± 1.0 1.6 ± 1.2 1.8 ± 1.1
Mean mSBI	Test Control Total	2.6 ± 0.7 2.7 ± 0.7 2.7 ± 0.7	1.5 ± 1.1 1.7 ± 1.1 1.6 ± 1.1	1.2 ± 1.1 1.0 ± 1.0 1.1 ± 1.1	1.2 ± 1.0 1.8 ± 1.1 1.5 ± 1.1	1.5 ± 1.1 1.0 ± 1.0 1.2 ± 1.2
mPlI	Test Control Total	2.0(1.0,2.0) 2.0(1.0,2.0) 2.0(1.0,2.0)	1.0 (0.0,2.0) 1.0(1.0,2.0) 1.0 (0.0,2.0)	0.0(0.0,1.0) 1.0(0.0,1.0) 1.0(0.0,1.0)	1.0(0.0,1.0) 1.0(0.0,1.0) 1.0(0.0,1.0)	1.0(0.0,1.5) 1.0(1.0,1.0) 1.0(0.0,1.0)
REC	Test Control Total	/ / /	0.0(0.0,1.0) 0.0(0.0,1.0) 0.0(0.0,1.0)	/ / /	0.0(0.0,1.0) 0.0(0.0,1.0) 0.0(0.0,1.0)	/ / /
Mean MBL, mm	Test Control Total	2.1 ± 1.8 3.0 ± 2.0 2.6 ± 1.9	/ / /	/ / /	1.7 ± 1.6 2.8 ± 2.0 2.3 ± 1.8	0.4 ± 0.6 0.2 ± 0.3 0.3 ± 0.5

Abbreviations: *PPD* peri-implant probing depth, *PPDd* deepest PPD; *mSBI* modified sulcus bleeding index, *mPlI* modified plaque index, *REC* mucosal recession, *MBL* marginal bone level

Δ showed the intragroup difference of every clinical parameter between baseline and each time point

**Table 4:** Intergroup comparison of Peri-Implant parameters at T2

Adjusted analysis^b^	Crude analysis^a^			Adjusted analysis^b^	
	Statistics		*P*	Statistics	*P*
Mean PPD	*t* = 2.16		**0.031**	t=-0.74	0.461
Mean mSBI	*t* = 4.31		**< 0.001**	t=-2.19	**0.028**
mPlI	*Z*=-1.88		0.060	Z=-0.59	0.550

Abbreviations: *PPD* peri-implant probing depth, *mSBI* modified sulcus bleeding index, *mPlI* modified plaque index

“^a^” analyzed using t-test; “^b^” adjusted through mixed-effects models. *P* < 0.05 are indicated by bold font

### Secondary outcomes

**Fig. 2:**
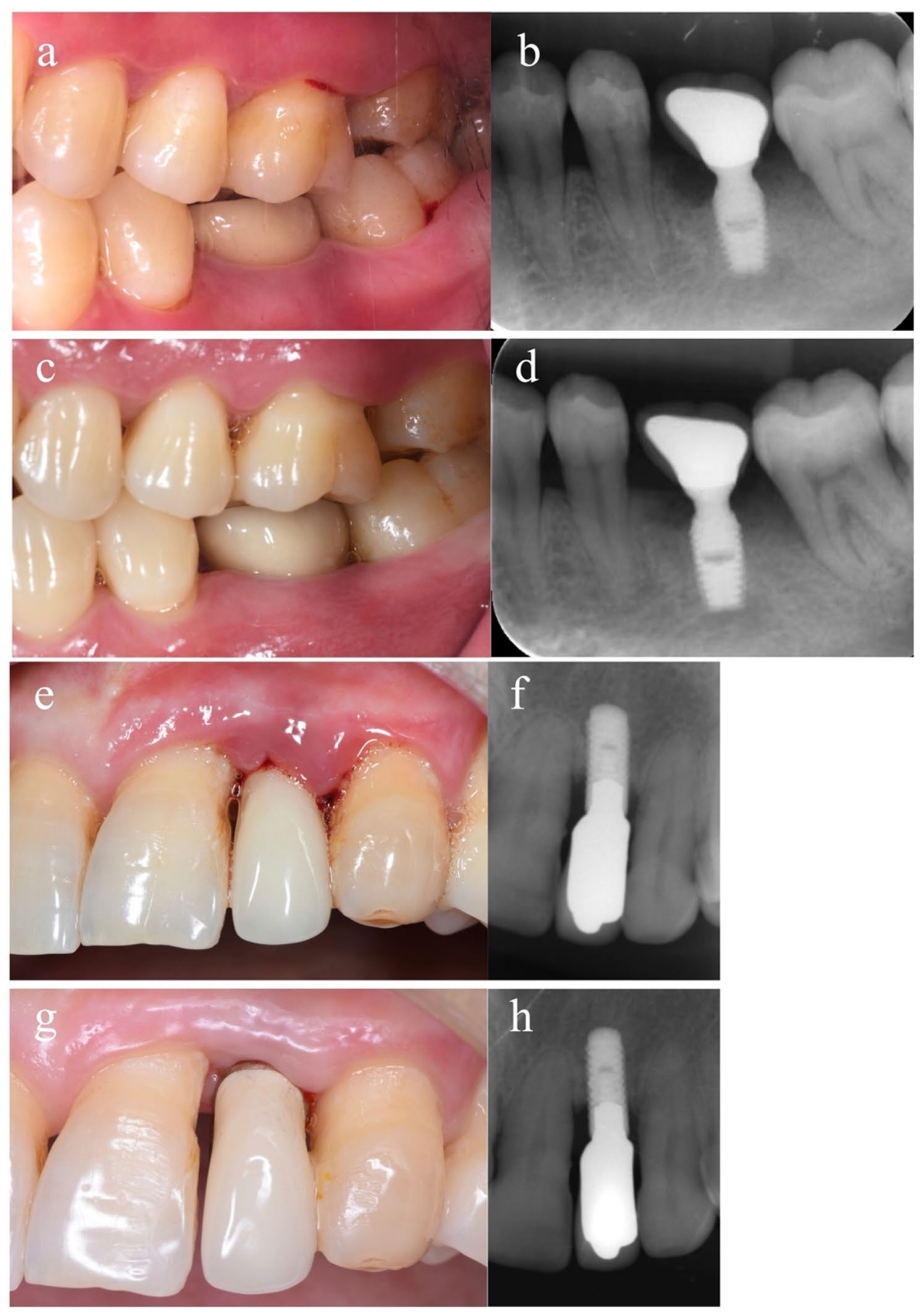
Clinical and radiographic images of the infected implant from both group.Peri-implantitis soft tissue inflammation and bone loss before non-surgical treatment from the test group (**a**&**b**) and control group (**e**&**f**);Inflammation resolution and bone level stabilization after the 3 months follow-up re-examination from the test group (**c**&**d**) and control group (**g**&**h**)

Both groups showed different degrees of improvement in clinical parameters. ​At T2, the mean mSBI decreased to 1.2 ± 1.0 and 1.8 ± 1.1 respectively, with a statistically significant reduction in the test group (P  < 0.05).​​ No significant differences were observed in the other parameters. Suppuration persisted in only one implant in the control group (from a baseline rate of 36.84%) but resolved completely in the test group (from a baseline rate of 47.37%) at T2. Only one implant in the test group met the defined treatment success criteria, versus none in the control group. Representative cases from both groups before and after treatment are shown in Figs. 2.​​​​.

### Periodontal clinical parameters outcomes

Table 5 demonstrates the improvement in periodontal parameters​ for full mouth and teeth adjacent to implants. Both groups exhibited statistically significant improvements in full-mouth PD and BoP% at T2.

**Table 5:** Periodontal clinical parameters at baseline(T0) and 3 months(T2) results

	Groups	T0	T2		ΔT0-T2
Periodontal clinical parameters					
Mean PD, mm	Test Control Total	3.5 ± 0.6 3.4 ± 0.6 3.5 ± 0.6	2.8 ± 0.3^*^ 3.0 ± 0.3^*^ 2.9 ± 0.3^*^		0.7 ± 0.5 0.5 ± 0.5 0.6 ± 0.5
BoP %	Test Control Total	25.9 ± 7.7 23.9 ± 6.7 24.4 ± 7.9	18.3 ± 7.6^*^ 19.3 ± 7.8^*^ 19.1 ± 7.4^*^		7.6 ± 6.5 4.6 ± 6.1 5.3 ± 6.7
Mean PD of adjacent teeth, mm	Test Control Total	3.3(2.9,4.9) 3.5(2.8,4.9) 3.4(2.9,4.9)	2.8(2.5,3.5)^*^ 2.8(2.7,4.0)^*^ 2.8(2.7,3,5)^*^		0.5(0.3,1.2) 0.4(0.0,0.8) 0.5(0.1,0.9)
BoP of adjacent teeth, %	Test Control Total	100.0(100.0,100.0) 83.3(50.0,100.0) 100(75.0,100.0)	66.7(50.0,100.0)^*^ 75.0(50.0,100.0)^*^ 75.0(50.0,100.0)^*^		25.0(0.0,50.0) 0.0(0.0,25.0) 0.0(0.0,25.0)

Abbreviations: *PD* probing depth, *BoP* bleeding on probing

Δ showed the intragroup difference of every clinical parameter between baseline and each time point

^*^ showed significant difference(*P*<0.05) for intragroup analysis using paired sampled t test or Wilcoxon-signed rank test

### Factors related to PPD outcome

Multivariate analysis results are presented in Table 6.​​ GEE model​ estimates revealed KMW​, PPD-T0​, mSBI-T2 and PD reduction of adjacent teeth ​were significant factors. Specifically, implants with KMW​ ≥ 2 mm were ​9.8 times more likely​ to achieve PPD-T2 ≤ 5 mm. Greater improvement in PD of adjacent teeth was associated with increased likelihood of achieving PPD-T2 ≤ 5 mm. Conversely, higher PPD-T0​​ and mSBI-T2 were associated with ​reduced likelihood​ of achieving PPD-T2 ≤ 5 mm.

**Table 6:** Generalized estimating equations model results of PPD-T2

				95% Confidence Interval					
Variables	Estimate	SE	Lower Limit		Upper Limit	Z		*P*	OR (95%CI)
Intercept	8.848	1.954	5.018		12.679	4.528		**< **0.001^*^	
PIKM									
< 2 mm									
≥ 2 mm	2.280	0.529	1.243		3.317	4.309		**< **0.001^*^	9.778(3.466 ~ 27.586)
Location									
Anterior tooth									
Premolar	-0.504	0.811	-2.093		1.086	-0.621		0.535	0.604(0.123 ~ 2.962)
Molar	-0.094	0.795	-1.651		1.464	-0.118		0.906	0.911(0.192 ~ 4.322)
Post-implant time									
< 5 years									
≥ 5 years	1.001	0.648	-0.270		2.272	1.543		0.123	2.721(0.763 ~ 9.698)
Percentage of PD-T2 ≥ 6 mm	-0.003	0.081	-0.161		0.155	-0.040		0.968	0.997(0.851 ~ 1.167)
mSBI-T2	-0.845	0.322	-1.476		-0.215	-2.628		0.009^*^	0.429(0.229 ~ 0.807)
MT	0.472	0.589	-0.683		1.626	0.801		0.423	1.603(0.505 ~ 5.086)
PPD-T0	-1.287	0.183	-1.646		-0.928	-7.024		**< **0.001^*^	0.276(0.193 ~ 0.395)
PD reduction of adjacent teeth	0.974	0.342	0.303		1.644	2.847		0.004^*^	2.648(1.354 ~ 5.177)
BI reduction of adjacent teeth	-0.135	0.359	-0.838		0.568	-0.377		0.706	0.874(0.433 ~ 1.764)

Abbreviations: *PIKM* peri-implant keratinized mucosa, *PD* probing depth, *mSBI* modified sulcus bleeding index, *MT* mucosal thickness, *PPD* peri-implant probing depth, *BI* bleeding index

^*^ showed significant difference(*P*<0.05)

Peri-implantitis soft tissue inflammation and bone loss before non-surgical treatment from the test group (a&b) and control group (e&f);

Inflammation resolution and bone level stabilization after the 3 months follow-up re-examination from the test group (c&d) and control group (g&h)

## Discussion

This randomized controlled clinical trial reports the 3-month clinical and radiographic outcomes of non-surgical treatment for peri-implantitis in patients with stage III/IV periodontitis.​​ Critical findings revealed that ​both groups exhibited clinical parameters improvement. The erythritol submarginal air-polishing group demonstrated statistically significant greater improvement in mSBI than the combined ultrasonic and manual instrumentation group. Furthermore, based on a limited sample size, several peri-implant and periodontal factors that may influence the PPD outcome were identified.

Previously, few studies have investigated the use of air-polishing alone for peri-implantitis, with only two reporting the results of erythritol air-polishing [11, 18, 19, 31–33]. Pan et al. used air-polishing for the treatment of early peri-implantitis, revealing that at the 3-month follow-up, the glycine group exhibited a 0.5 mm reduction in PPD and the erythritol group exhibited a 1.0 mm decrease, accompanied by superior long-term anti-inflammatory efficacy (lower IL-1β level at 6 months) [19]. Hentenaar et al. observed only minimal improvements in BoP%, suppuration%, PPD (0.3–0.5 mm reduction), and MBL (0–0.1 mm reduction) after submarginal erythritol air-polishing at 3-month follow-up [18]. Renvent et al. similarly reported no statistically significant changes in clinical or radiographic parameters when comparing glycine air-polishing with Er: YAG laser therapy [11]. John et al. documented a mere 0.4–0.5 mm PPD improvement after 12 months of glycine submarginal air-polishing, but with a greater reduction in BoP% (41.2 ± 29.5%) than mechanical debridement alone (16.6 ± 33.4%) [32]. Notably, Merli et al. found that glycine air-polishing resulted in negligible PPD improvement (0.1 ± 0.8 mm at 6 months) and substantially higher pain levels [33]. Our study is the first to compare erythritol air-polishing and combined instrumentation debridement in patients with severe periodontitis, demonstrating greater PPD improvement, which may be attributable primarily to the higher baseline severity of periodontal and peri-implant inflammation in our cohort (mean PPD 6.0 mm vs. 4.2 mm in Hentenaar et al.’s study). Elevated peri-implant pocket depth correlates with significant shifts in the submucosal microbiome toward dysbiosis [34], potentially explaining the greater therapeutic response in advanced cases. Similarly, our MBL gain exceeded the 0.1 mm threshold historically reported, suggesting that severe peri-implantitis may have greater regenerative potential after non-surgical intervention.

A systematic review reported that non-surgical treatment success rates without antibiotics range from 15% to 55%, though criteria varied across studies [35]. In our study, according to the success criteria for non-surgical treatment defined by the 2023 EFP Clinical Practice Guideline, the test group achieved a low success rate of 2.6%. Nevertheless, considering only resolution of PPD and suppuration (excluding BoP), the T2 success rate was 47.4% (9 out of 19 implants successful in each group), suggesting that clinicians should maintain attention to controlling soft tissue inflammation. Using the same criteria, Laleman et al. reported a similarly low 10.5% success rate after 24 months of non-surgical treatment [36]. By contrast, Hentenaar et al. excluded BoP and used a stricter PPD threshold(≤ 4 mm), reporting a success rate of 17.3% at 3 months after treatment.

The consensus established at the 2015 Eighth European Workshop on Periodontology concluded that air-polishing with glycine powder effectively removes peri-implant biofilm deposits and demonstrates advantages in resolving inflammation [37]. Systematic evidence further corroborates glycine air-polishing’s advantage in BoP improvement compared with curette scalers or Er: YAG lasers [15, 38]. Our findings substantiate that erythritol air-polishing surpassed combined instrumentation in resolving peri-implant soft tissue inflammation, as evidenced by significantly greater reductions in mSBI. This is consistent with John et al.‘s study [32].

Mechanical debridement instruments used for treatment may cause alterations to the implant surface. Softer plastic materials seem to have a limited debridement power, as they may not completely remove the biofilm. In addition, plastic remnants could remain after instrumentation [39]. On the other hand, ultrasonic scalers with metal tips and metal curettes have been proven to efficiently remove biofilm, but may create scratches on the implant surfaces, especially if used improperly [40, 41]. By contrast, erythritol polishing provided better cleaning efficacy than curettes and ultrasonic scalers, while causing minimal damage to the titanium surface for deep‑threaded implants. After treatment, the amount of titanium particles released into the surrounding soft tissues was the lowest, further reducing the biological risks [42]. Most previous studies have focused on glycine powder, a biocompatible non-essential amino acid with very fine particle size (25 μm) and a hardness lower than titanium. In comparison with glycine powder, erythritol powder exhibited stronger antibacterial activity and a greater ability to inhibit biofilm formation [43], indicating that it may provide potential benefits for peri‑implant debridement.

Furthermore, this study identified that deeper PPD-T0 was associated with a lower probability of achieving treatment success (PPD ≤ 5 mm) at 3 months, highlighting inadequate debridement capability in deep pockets as a key limitation of non-surgical peri-implantitis therapy. Higher mSBI-T2 was also associated with a lower treatment success of PPD. Consistent with prior research demonstrating a significant positive association between BoP and PPD around dental implants [44, 45], our results confirm this relationship. Meanwhile, PD reduction of adjacent teeth positively associated with PPD treatment efficacy. Yan et al. reported a significant correlation in pathogenic bacteria between implants and adjacent teeth, concluding that adjacent teeth may serve as microbial reservoirs for peri-implant bacteria [46]. Previous studies have established that peri-implantitis compromises periodontal conditions of adjacent teeth and diminishes the efficacy of non-surgical therapy [47, 48]. Our findings further demonstrate a reciprocal influence between implants and adjacent teeth. Consequently, patients with periodontitis require comprehensive periodontal and peri-implant maintenance therapy both before implant placement and after prosthetic restoration to prevent the development of peri-implant diseases. Inadequate peri-implant keratinized mucosa (PIKM) is associated with a higher prevalence of peri-implantitis, biofilm accumulation, soft-tissue inflammation, mucosal recession, marginal bone loss, and greater patient discomfort [49]. In this study, adequate PIKM (≥ 2 mm) was confirmed as a significant predictor of reaching PPD ≤ 5 mm (OR = 9.778, *P* < 0.001) — aligning with Fons-Badal et al.‘s findings [50]. Moreover, evidence indicated that implants with adequate PIKM exhibit significantly greater improvement in BoP and more effective in achieving success following treatment of peri-implant mucositis [51]. This mechanism was biologically plausible due to its capacity to enhance mechanical resistance of peri-implant tissues and facilitate effective plaque control adjacent to implant restorations [52]. Inadequate PIKM is established not only as a risk factor for peri-implant disease prevalence [53] but also as an influencing factor on non-surgical treatment outcomes.

The 3-month observation period in this study aligns with the recommended re-evaluation interval following non-surgical peri-implantitis therapy outlined in guidelines [30]. Partido et al. reported the clinical and microbial changes of peri-implant mucositis induced by non-surgical treatment were sustained for a maximum of 3 months [54]. Most implants failing to meet therapeutic success criteria should be actively managed with either repeat non-surgical debridement or surgical intervention [18].

Currently, case reports have indicated that emphysema may occur as a complication following air‑polishing [20–22], which should alert clinicians when using this approach. No emphysema occurred in the enrolled patients, likely due to strict compliance with indication criteria and standardized procedures. Air‑polishing should be strictly contraindicated in sites adjacent to unhealed extraction sockets or areas with acute inflammation.

This study has several limitations. First, patient-related outcomes (PROs) such as pain perception or functional comfort were not assessed, which could provide critical insights into treatment acceptability. Second, external validity is limited due to stringent eligibility criteria (systemically healthy non-smokers). Future large-scale multicenter studies are warranted to validate these results.

## Conclusion

In this 3-month follow-up study, both non-surgical therapies demonstrated efficacy in improving clinical parameters of peri-implantitis among patients with stage III/IV periodontitis. Erythritol submarginal air-polishing exhibited significantly greater improvement of soft tissue inflammation. Critically, therapeutic outcomes of peri-implantitis were significantly influenced by the peri-implant keratinized mucosa width and probing depth reduction in adjacent teeth, underscoring that clinicians should manage periodontitis concurrently when treating peri-implantitis.

## Data Availability

Clinical data cannot be shared publicly to protect patient confidentiality. De-identified data may be requested from the Ethics Committee of the Peking University School and Hospital of Stomatology (contact: keyanchuethics@163.com) and corresponding author (contact: [shidong@pkuss.bjmu.edu.cn](mailto: shidong@pkuss.bjmu.edu.cn) ) for research meeting ethical standards.

## Abbreviations

​BI​: Bleeding Index
​BoP​: Bleeding on Probing
​CONSORT​: Consolidated Standards of Reporting Trials
​EMS​: Electro Medical Systems
​FMPS​: Full-Mouth Plaque Score
​GEE​: Generalized Estimating Equations
​ICC​: Intraclass Correlation Coefficient
​KMW​: Keratinized Mucosa Width
​MBL​: Marginal Bone Level
​MCID​: Minimal Clinically Important Difference
​mPlI​: Modified Plaque Index
​mSBI​: Modified Sulcus Bleeding Index
​MT​: Mucosal Thickness
​PCCL​: Peri-Implant Clinical Crown Length
​PD​: Probing Depth
​PIKM​: Peri-Implant Keratinized Mucosa
​PPD​: Peri-Implant Probing Depth
​PROs​: Patient-Related Outcomes
​REC​: Mucosal Recession
